# Implementation and sustainment of virtual reality stroke workflow training for physician trainees at comprehensive stroke centres: a quantitative and qualitative study

**DOI:** 10.1186/s12909-024-06438-3

**Published:** 2024-12-19

**Authors:** Steven Maltby, Joshua J. Mahadevan, Neil J. Spratt, Carlos Garcia-Esperon, Murielle G. Kluge, Christine L. Paul, Timothy J. Kleinig, Christopher R. Levi, Frederick R. Walker

**Affiliations:** 1 Centre for Advanced Training Systems, The University of Newcastle, Callaghan, NSW Australia; 2 School of Biomedical Sciences & Pharmacy, College of Health, Medicine & Wellbeing, The University of Newcastle, Callaghan, NSW Australia; 3 Hunter Medical Research Institute, New Lambton Heights, NSW Australia; 4 Department of Neurology, Royal Adelaide Hospital, Port Road, Adelaide, SA Australia; 5 Faculty of Health and Medical Sciences, The University of Adelaide, Adelaide, SA Australia; 6 Department of Neurology, John Hunter Hospital, New Lambton Heights, NSW Australia; 7 School of Medicine and Public Health, College of Health, Medicine & Wellbeing, The University of Newcastle, Callaghan, NSW Australia; 8 John Hunter Health & Innovation Precinct, New Lambton Heights, NSW Australia

**Keywords:** Virtual reality, Technology, Medical education, Stroke management, Stroke workflow

## Abstract

**Background:**

Variation in stroke treatment metrics highlight a need for approaches to improve clinical processes. Training interventions can improve outcomes, but Australian physician trainees do not currently receive formal process-directed stroke training. Virtual reality (VR) stroke workflow training has proven acceptable, usable, useful and feasible in trial contexts, but how to integrate VR training into physician training remains unclear. The current study sought to document stroke staff perceptions of existing training and assess implementation of routine VR training at comprehensive stroke centres, outside of a trial context.

**Methods:**

Training was delivered to physician trainees via individual sessions or facilitated group workshops depending on the hospital site. VR usage data was captured automatically via Wi-Fi. Survey responses from both trainees and training staff were collected, with statistical comparisons performed for matching questions in pre- and post-training surveys. Themes identified in open-ended survey responses were enumerated and reported.

**Results:**

Forty-two TACTICS VR training sessions were logged at 2 hospitals between May 2022 and October 2023. Trainees reported receiving low amounts of prior formal stroke training; both trainees and training staff identified unmet needs and barriers to existing training. VR users (*n* = 30) provided positive feedback on VR hardware, software design, user experience, content, educational value and delivery approach (mean scores 3.9 to 4.7; 1 = strongly disagree, 5 = strongly agree). VR training improved confidence in: knowledge of acute stroke assessment / treatment (post-training vs. pre-training = 4.0±0.7 vs. 2.9±1.0; *P* < .0001), ability to effectively assess / treat stroke (4.0±0.6 vs. 3.1±1.0; *P* < .0001), ability to optimally communicate with colleagues (4.1±0.6 vs. 3.3±1.0; *P* < .001), understanding of workflow practices (4.3±0.6 vs. 3.2±1.2; *P* < .0001), ability to make improvements (4.1±0.8 vs. 3.0±1.2; *P* < .0001) and awareness of local stroke management criteria / processes (4.1±0.8 vs. 3.6±1.1; *P* < .01). Respondents suggested enhancements in funding, access, awareness, training populations and delivery modality to improve training sustainment.

**Conclusions:**

VR stroke workflow training was perceived by trainees and training staff as feasible, acceptable, usable, useful and positively impacted stroke training. Respondents endorsed future use of VR training to support training at comprehensive stroke centres and identified aspects for improved future integration.

**Supplementary Information:**

The online version contains supplementary material available at 10.1186/s12909-024-06438-3.

## Background

Stroke is a medical emergency, which contributes to significant patient and healthcare system burden. Treatment is time-critical, with delays (in a typical hemispheric stroke) resulting in loss of 1.9 million brain cells per minute of delay [1]. This can cause substantial patient harm and socioeconomic cost − 0.77 quality-adjusted life years and >$10,000 AUD in associated costs for each hour of delay [2, 3]. Australian audit data highlights multiple gaps in hyper-acute stroke management with only 10% of patients receiving thrombolysis treatment (despite an estimated 20% being eligible) and 29% of the patients receiving thrombolysis treatment within the target timeframe of < 60-minutes [4]. While many factors likely contribute to these metrics, we propose physician and physician-trainee education as a key aspect that can be targeted to improve stroke care. It will be critical to identify education approaches that can be implemented within the constraints of current resourcing and training frameworks.

Stroke training is integrated across multiple stages of emergency and internal medicine physician training. In Australian medical schools, focus is primarily on foundational knowledge (e.g. neuroanatomy, stroke subtypes and stroke therapeutics) but little emphasis is placed on practical approaches to improve treatment speed. During basic internal medicine physician training, trainees gain direct case exposure during neurology or general medicine rotations. Curriculum includes stroke knowledge, rehabilitation and appropriate referral [5]. In emergency medicine training, curriculum includes familiarisation with the function of stroke units and their use in relation to patient flow in emergency departments [6]. However, individual trainee experiences vary depending on case exposure and local education activities, which are highly dependent on allocated rotations. This phase will be the only direct practical stroke training many physicians receive before dispersing across the health system. For physicians that enter advanced training in clinical neurology, exposure to hyperacute stroke is variable and informed by local case-mix and quality improvement processes. During this phase, the FRACP curriculum outlines high-level skills in structured assessment (e.g. NIHSS scale), diagnosis, treatment, rehabilitation and prophylaxis [7]. Continuing professional education is largely ad hoc, including self-directed study and participation in seminars or conferences. Australian healthcare staff currently receive limited practical and case-based procedural training in stroke (< 2/3 of physicians and nurses report interactive or competency-based training) [8]. Training interventions can improve stroke workflow processes [9–11], albeit with relatively minor improvements observed when implementing individual interventions (e.g. <10%) [12]. To date, limited studies have assessed approaches applying simulation for stroke workflow training [13].

Virtual reality (VR) is increasingly being applied for workplace-based training, including for tertiary education [14, 15], defence [16, 17] and healthcare [18–24]. VR technology can provide realistic and engaging training, with an increased sense of user presence in the training environment, immersion and physiological responses compared to 2D delivery [25]. Multiple studies have assessed VR training for a range of medical conditions and healthcare professional populations, consistently reporting improvements in trainee engagement, confidence and skills acquisition [18–24]. Specific to stroke training, VR applications have primarily targeted patients (e.g. for limb rehabilitation) rather than healthcare professionals [26, 27]. The TACTICS VR training platform provides hyperacute stroke workflow training from a physician perspective [28–31]. Previous studies demonstrated training was acceptable, useful, feasible and had positive perceived training impacts [28, 29], and when delivered as a part of a broader implementation package was associated with reduced stroke treatment times and increased thrombolysis rates [31]. However, these studies were conducted in structured trial contexts, did not specifically target a physician trainee population and no feedback was collected from staff delivering stroke training. More broadly, very few studies have assessed practical aspects and approaches to sustain health education interventions over the long-term in any disease context [32].

The current study sought to document stroke staff perceptions of training and assess VR training at comprehensive stroke centres outside of a trial context including: exploring enablers, barriers and areas of unmet need in existing stroke training; assessing implementation of VR training within routine training at comprehensive stroke hospitals; assessing training for a physician trainee population; and collecting data from a training staff perspective. The primary aim of the current study was to determine the feasibility, acceptability, usability and perceived training effects of implementing VR stroke workflow training within routine training activities at 2 comprehensive stroke hospitals. Secondary aims included (1) assessment of attitudes towards existing stroke training from both a trainee and training staff perspective and (2) collection of feedback to inform future adaptation of VR training modules to scale, integrate and sustain training in the future.

## Methods

### Design

Self-reported data was collected both automatically within the VR headset for all trainee participants (quantitative) and via surveys for both trainees and training staff (quantitative and qualitative).

### Setting

The current study was conducted at 2 comprehensive stroke teaching hospitals, located in New South Wales (NSW) and South Australia (SA), respectively. Both sites participated in the original TACTICS trial, which included deployment of the 1st TACTICS VR – Hyperacute Stroke Management training module [28, 30] in October 2019 (NSW) and August 2020 (SA).

### Procedures

Different implementation approaches were applied at each site, informed by local constraints and existing training programs. A single Oculus Quest 2 headset was deployed at the SA site. The SA research lead was initially trained to use the VR headset and additional support was available on an as-needed basis, but was not required. Deployment at the NSW site occurred using multiple headsets to support group training in 1-hour face-to-face workshops. A brief face-to-face orientation was provided by a research team member and VR training was followed by a debriefing with experienced stroke clinicians. Findings were very similar across sites and were pooled to improve the overall generalisability of findings, independent of the specific implementation approach applied. The research team met regularly (approximately monthly) to review VR training usage and discuss adjustments to optimise approach iteratively across trainee cohorts.

### Participants

The target training audience at both sites were physician trainees on rotation, including basic physician and advanced trainees (corresponding to approximately 3 to 7 years post-graduate). Survey feedback was also sought from training staff involved in deployment of VR training.

### Intervention

The TACTICS VR – Hyperacute Stroke Management and Stroke Telehealth modules have previously been described in detail [28–31]. Briefly, VR users work through a real-world stroke case from initial notification, through assessment, advanced imaging, consent and treatment. Users interact with the VR environment, actively making decisions and receiving real-time feedback. All interactions and training times are automatically transmitted via Wi-Fi to a password-protected cloud-based database [28, 29]. Minor adaptations to the TACTICS VR modules were made in the current study including addition of the SA location for demographics capture and a note that minor aspects of workflow differ from local processes (e.g. timing of telehealth calls).

### Data collection & measures

Data was collected from 10 May 2022 through 05 October 2023 inclusive.

In the VR training module, basic demographics information was automatically collected via user prompts (location, training date, total training time, penalties). This data is presented as “user data” throughout this manuscript.

Trainees were invited to complete surveys before (“trainee pre-survey”) and after (“trainee post-survey”) VR training. Upon completion of the implementation phase, staff involved in delivery of VR training were invited to complete surveys online (“training staff survey”). Surveys were drafted with input from research team members and adapted from previous studies (full question list and responses available in Supplementary Materials). Survey design was informed by the Technology Acceptance Model [33–35], focused on perceived acceptability, usability, usefulness and training impacts [36, 37]. Surveys were generated in Microsoft Forms and accessed via QR code, or available as paper versions. Total survey length was minimised in recognition of the time constraints of the target audience. All survey data are referred to as “survey response data” and/or “respondents” throughout manuscript.

### Statistical analysis

All data from both sites was pooled and data from trainees and training staff are reported separately. Statistical analysis was performed using Prism v.9 (GraphPad, USA). Quantitative survey and VR training usage data is presented as mean ± standard deviation (SD) or absolute number of responses as indicated in the text and every response was included. Comparisons for questions repeated in pre- and post-training surveys were analysed using t-tests, adjusted for multiple comparisons. *P* < .05 were considered statistically significant.

No formal analysis of qualitative data (i.e. open-text format survey questions) was performed. Points which were repeatedly mentioned were identified and counted by the study investigators for each question and example quotes are provided in the manuscript text (a full list of responses is provided in Supplementary Materials).

## Results

### Iterative VR training deployment strategy

At the SA site, the implementation approach was incrementally adapted across the study period across 5 consecutive trainee cohorts. For Cohort 1 (May 2022; n = ∼ 12), an overview of VR training was provided, and training made available as an optional supplement to existing training. External events were noted that may have affected uptake, including increased workload due to an influenza outbreak and medical exam leave resulting in staggered initiation. For Cohort 2 (August 2022; n = ∼ 11), VR training was included in the department orientation and was considered mandatory. Training was also offered more broadly to stroke department staff via email. For Cohort 3 (October 2022; n = ∼ 8), VR training was formally introduced as mandatory and documented in the training report at rotation completion. External events were again noted, including staffing changeover during the end-of-year period with limited staffing and supervisor availability. For Cohort 4 (February 2023; n = ∼ 10), VR training was also mandatory, and the VR headset was moved to the stroke department office with oversight by a research trial nurse. Reminders were provided at departmental meetings and direct supervisors and trainees were actively encouraged to engage with training during downtime. For Cohort 5 (May 2023; n = ∼ 8), VR training was introduced during the orientation, was mandatory and efforts were made to provide trainees with protected training time. It was noted that 2 trainees were infected with Covid-19 early in the rotation, resulting in limited training uptake in the first month.

At the NSW site, VR training was provided during 2 discrete scheduled workshops with multiple headsets for parallel group delivery (*n* = 4 trainees per session). The group delivery format provided an opportunity for trainees to discuss the VR training with experienced stroke clinicians during debriefing, to consolidate learnings and consider how specific aspects align with local processes. The format also provided an opportunity for additional group education (e.g. updates on local research trial recruiting).

### Survey respondent demographics

**Fig. 1:**
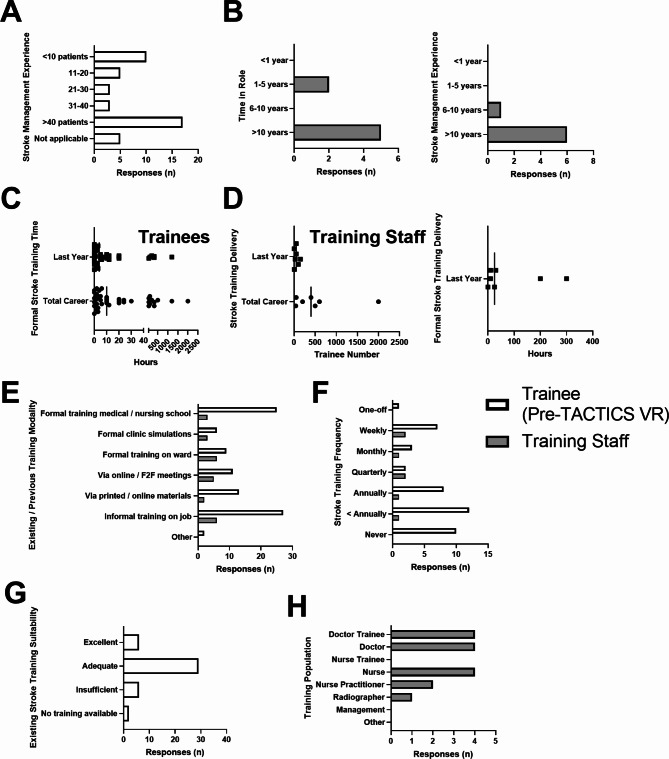
Trainees have a range of previous stroke management and existing stroke training is diverse and variable. (**A**) Trainee and (**B**) training staff stroke management experience, (**C**/**D**) formal stroke training time / delivery, (**E**) existing stroke training modality, (**F**) training frequency, (**G**) perception of training suitability and H) target training populations. Data presented as number of responses or individual data points with median indicated. White bars = trainee pre-VR survey; grey bars = training staff survey

All VR users were invited to complete surveys and 43 pre-training and 30 post-training responses were received. Respondents were based in Acute Stroke / Neurology (*n* = 36) or Other (*n* = 7; e.g., trainee, student, intern). The majority were doctors (38), with 1 nurse and 4 “Other”. Twenty respondents identified as being in a training program (No = 23). Respondents had a range of previous experience in stroke management, with 10 having cared for / treated ≤ 10 patients and 17 > 40 patients (Fig. 1A). A subset of respondents indicated they were prone to motion sickness (*n* = 12), when asked a binary question (no = 31).

After the VR implementation phase, 7 training staff provided survey feedback. All respondents worked in Acute Stroke / Neurology and included doctors (4) and nurses (3). Training staff had varying time in the role (5 = > 10-years, 2 = 1–5 years) and extensive experience in stroke management (6 = > 10 years, 1 = 6–10 years; Fig. 1B).

### Existing stroke training and perceived unmet need

Trainees reported a range of formal stroke training time over their career (median = 10 h; min = 0, max = 2000) and in the previous year (median = 4 h; min = 0, max = 1200; Fig. 1C). Training staff had extensive experience delivering stroke training, in terms of the total trainee numbers over their career (median = 400; min = 40, max = 2000) and in the previous year (median = 50; min = 4, max = 150) and training time delivered in the previous year (median = 30; min = 0, max = 300; Fig. 1D).

When queried about previous and existing stroke training, both trainees and training staff indicated training occurred via a range of modalities (Fig. 1E) and formal training frequency varied, ranging from never or one-off to weekly (Fig. 1F). Trainees had varying views of current stroke training suitability, ranging from excellent (*n* = 6), adequate (29), insufficient (6) or no training available (2; Fig. 1G). Training staff trained multiple healthcare professional populations, with doctor trainees (*n* = 4), doctors (4) and nurses most common (4; Fig. 1H).

Trainees and training staff were asked open-ended questions regarding enablers and barriers to existing stroke training (all responses provided in Supplementary Materials). Trainees highlighted enablers as: on-site / staff-led training (*n* = 18; e.g. “consultant led”), formal training (*n* = 10; e.g. “formal orientation and training”), resources (*n* = 5; e.g. “online resources and protocols”), funding (*n* = 1) or no enablers (*n* = 3; e.g. “Nil”, “N/A”). Training staff provided similar responses, including formal training activities (*n* = 3; e.g. “formal training at start of junior staff rotations”), staff / on-site training activities (*n* = 3; e.g. “good relations with ED and other teams to be trained”) and resources (*n* = 2; e.g. “formalised training resources”).

When asked about barriers or areas of unmet need trainees primarily noted: time issues (*n* = 9; e.g. “too busy to engage”), staffing (*n* = 6), stroke process complexity and practicality (*n* = 5; e.g. “training of stroke patient flow at a systems levels”), resources (*n* = 3), funding (*n* = 2), indicated there were no barriers (*n* = 6) or were unsure (*n* = 4). Training staff highlighted similar barriers including: time (*n* = 4; e.g. “time constraints with clinical workload”), staffing (*n* = 4; e.g. “lack of dedicated staff to help organise”) and resources (*n* = 2).

Before VR training, when asked what they hoped to learn, trainees highlighted aspects of stroke management (*n* = 22; e.g. “how to manage code stroke”), VR technology (*n* = 7; e.g. “simulated learning experience”) or general enthusiasm (*n* = 2; “fun experience”).

### Previous VR experience and perceptions of VR for medical training

**Fig. 2:**
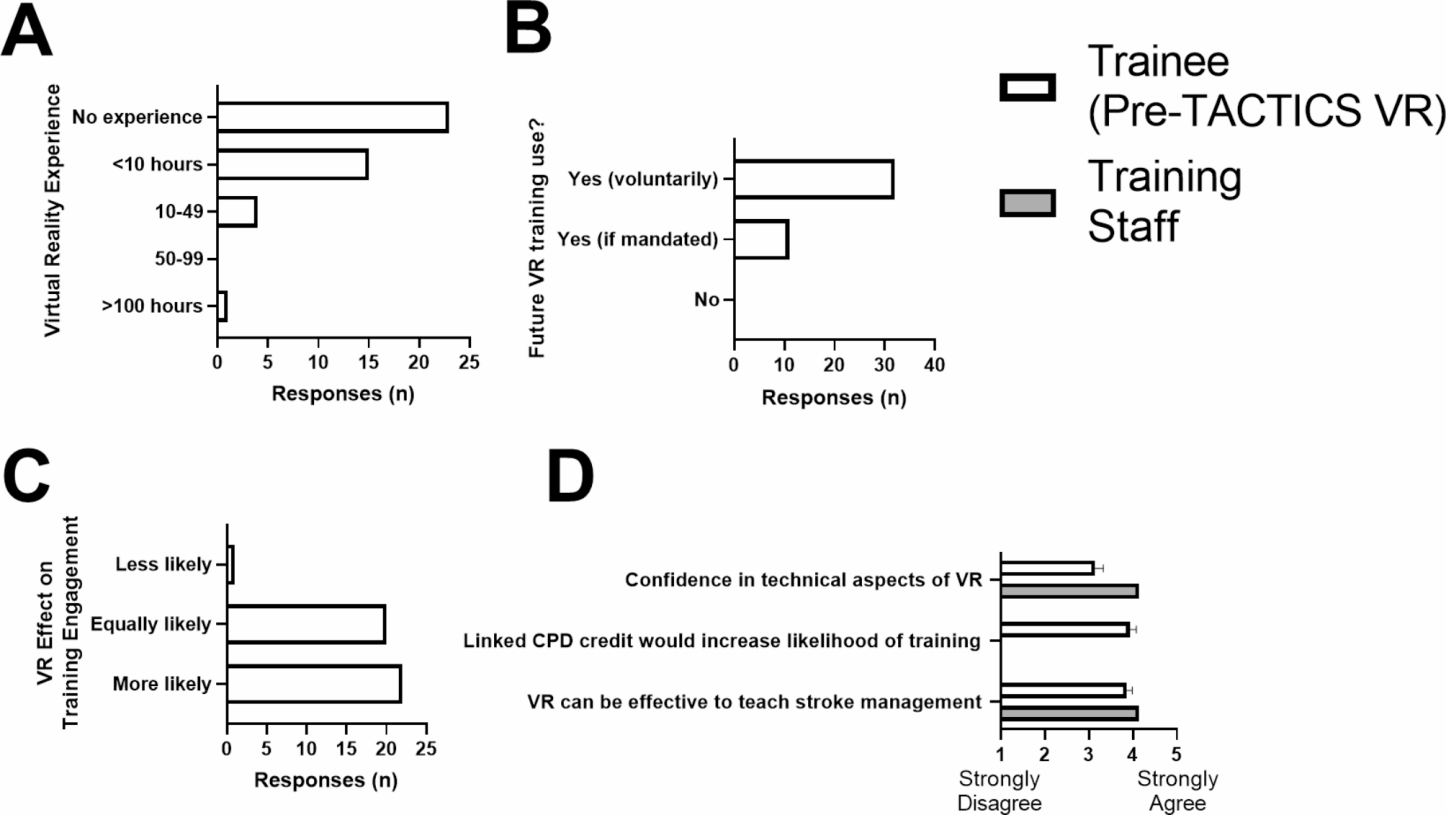
Respondents had limited previous VR experience but positive attitudes to VR training. Previous experience with (**A**) virtual reality in any context and perceptions of (**B**) future use of VR training if available, (**C**) effect of available VR training on future engagement and (**D**) attitudes to VR training. Data presented as number of responses or mean±SEM; 1 = strongly disagree, 5 = strongly agree. White bars = trainee pre-VR survey; grey bars = training staff survey. CPD = continued professional development

Trainee respondents had relatively little prior experience with VR technology (Fig. 2A). Three trainees had previously used TACTICS VR (1 = Hyperacute module; 2 = uncertain of module; 36 = No). Trainees all indicated they would use VR training in the future if it was available (32 voluntarily; 11 if mandated / required; 0 would not use; Fig. 2B). Twenty-two were more likely to engage with stroke training in the future if VR training was available (20 equally likely, 1 less likely; Fig. 2C). Both trainees and trainers agreed that VR can be effective to teach stroke management (trainees 3.9±0.8; training staff 4.1±0.7; 1 = strongly disagree, 5 = strongly agree) and were moderately confident in managing technical aspects (trainees 3.1±1.2; training staff 4.1±0.7; Fig. 2D). Further, trainees agreed that linking continuing professional development credits to training would increase their likelihood to use the technology (3.9±1.0; Fig. 2D).

### TACTICS VR training usage & user feedback

A total of 39 sessions were logged on TACTICS VR – Hyperacute Stroke Management (SA site = 31 sessions; NSW site = 8). At the SA site this included 6, 6, 5, 6 and 8 sessions across each of Cohorts 1–5, respectively (approximately 60% uptake across the estimated 49 trainees). Mean VR session time was 21 min 04 s ±5 min 11 s (mean±SD), equating to a total of 13 h 41 min. User accuracy was 84%±8% with an average of 24±17 min in time penalties. An additional 3 sessions were logged on TACTICS VR – Stroke Telehealth at the SA site, with mean session time of 12 min 48 s (total training = 38 min). User accuracy was 88% with 18 min in time penalties.

**Fig. 3:**
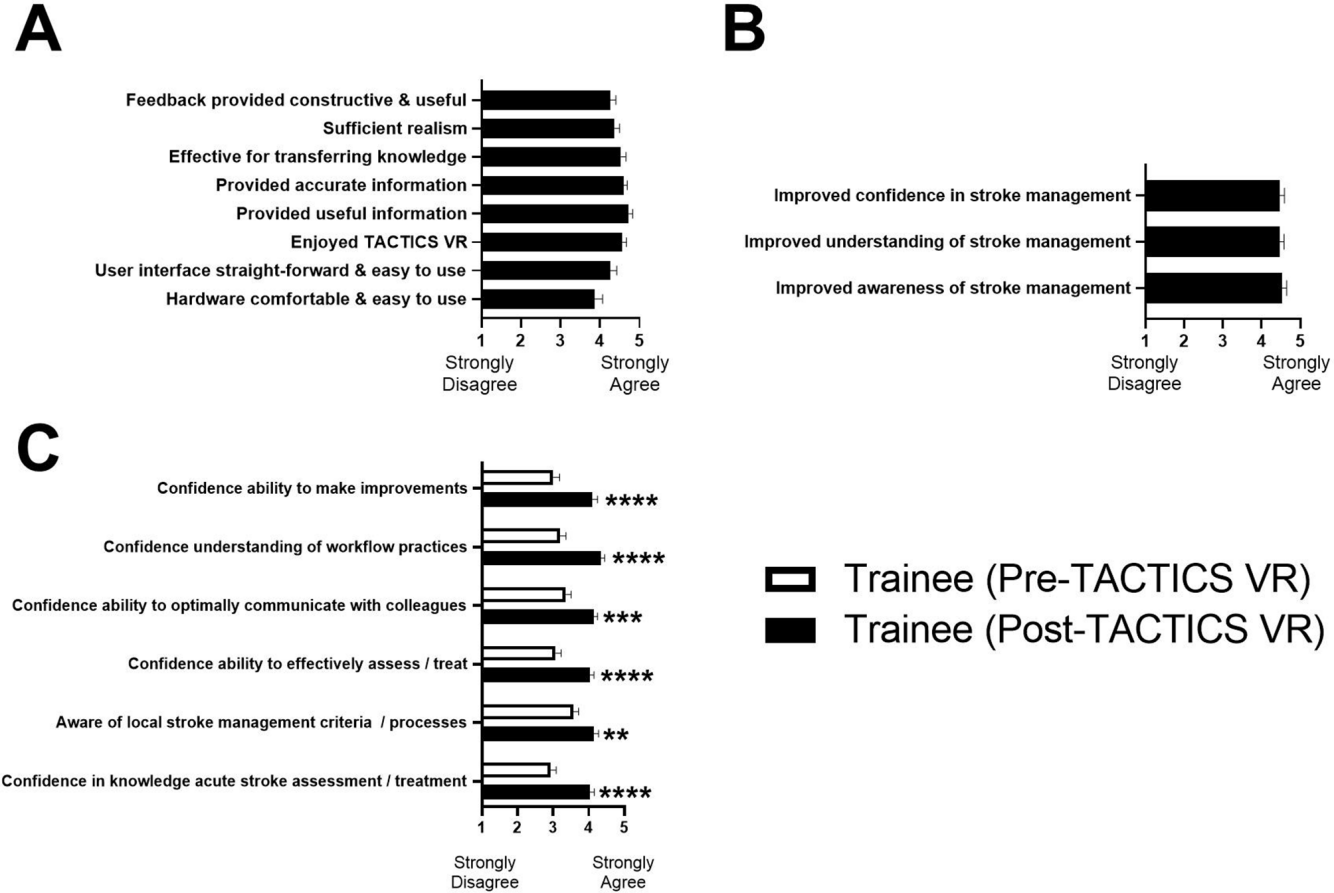
Trainees provide positive feedback on VR training and perceived training benefits, while training staff have relatively low confidence in new staff skills. (**A**) Survey feedback on hardware, content and feedback, (**B**) effects of training and (**C**) pre- versus post-training response comparisons for matched questions. Data presented as mean±SEM; 1 = strongly disagree, 5 = strongly agree. **P < .01, ***P < .001, ****P < .0001. White bars = trainee pre-VR survey; black bars = trainee post-VR survey

In post-training surveys, trainees provided positive feedback on a range of statements relating to hardware, software design and user experience, content, educational value, delivery approach and feedback (mean scores 3.9 to 4.7; 1 = strongly disagree, 5 = strongly agree; Fig. 3A). Further, trainees agreed that TACTICS VR improved their awareness, understanding and confidence in stroke management (4.5±0.6 for each statement; Fig. 3B). Twenty-six trainees and all 7 training staff believed TACTICS VR should be used in the future (trainees 3 = unsure, 1 = no). Further, 27 trainees and 5 training staff endorsed development of additional modules (trainees 3 = no; training staff 1 = no). In open-format questions, trainees indicated future modules should include: other stroke cases (*n* = 12; e.g. “stroke management with added twists or complications”), stroke mimics (*n* = 2), specific aspects of stroke assessment (*n* = 2; e.g. “completing a NIHSS”) and non-stroke clinical areas (*n* = 3; e.g. “sepsis, STEMI”). Training staff agreed that additional modules would be useful including: other aspects of stroke management (*n* = 4; e.g. “general stroke care”) and other clinical needs (*n* = 1; “pre-hospital”).

Twenty-six trainees reported no issues during VR training, while 4 reported issues related to aspects beyond the specific training content: time availability (*n* = 3; e.g. “difficult to secure time while managing ward duties”), hardware (*n* = 2; e.g. “no issues but difficult to adjust headset suitably for clear vision”), user error (*n* = 1) and funding (*n* = 1). Four training staff reported no issues, while 3 also reported issues external to specific VR content: engagement (*n* = 2; e.g. “difficult to motivate them to participate initially”) and training time (*n* = 2). When prompted, 6 trainees indicated they experienced mild motion sickness but were able to complete training (24 = no motion sickness; 0 motion sickness that required training discontinuation).

Trainee pre- and post-training surveys included matched questions to allow statistical comparisons of perceived training impact. Following VR training, trainees were significantly more likely to agree with each of the matched statements including: confidence in knowledge of acute stroke assessment / treatment (post-training vs. pre-training = 4.0±0.7 vs. 2.9±1.0; strongly agree = 5, strongly disagree = 1; *P* < .0001), confidence in ability to effectively assess / treat stroke (4.0±0.6 vs. 3.1±1.0; *P* < .0001), confidence in ability to optimally communicate with colleagues (4.1±0.6 vs. 3.3±1.0; *P* < .001), confidence in understanding of workflow practices (4.3±0.6 vs. 3.2±1.2; *P* < .0001), confidence in ability to make improvements (4.1±0.8 vs. 3.0±1.2; *P* < .0001) and increased awareness of local stroke management criteria / processes (4.1±0.8 vs. 3.6±1.1; *P* < .01; Fig. 3C).

In post-training surveys, when asked what elements of the TACTICS VR training module were most beneficial, trainees primarily referred to: user experience / content (*n* = 18; e.g. “feedback about choices made and pressure with [stroke / neuron] clocks at the top of the screen”) and VR modality (*n* = 8; e.g. “realism”). When asked what elements could be improved respondents highlighted: specific content (*n* = 10; “more education about treatment decision process”), user experience (*n* = 8; “length of explanations”), hardware (*n* = 2; “more comfortable headset”) and more scenarios (*n* = 1), while one respondent indicated the question was not applicable (“honestly can’t think of much, it was an excellent scenario”).

### Feedback on TACTICS VR deployment & sustainment approach

**Fig. 4:**
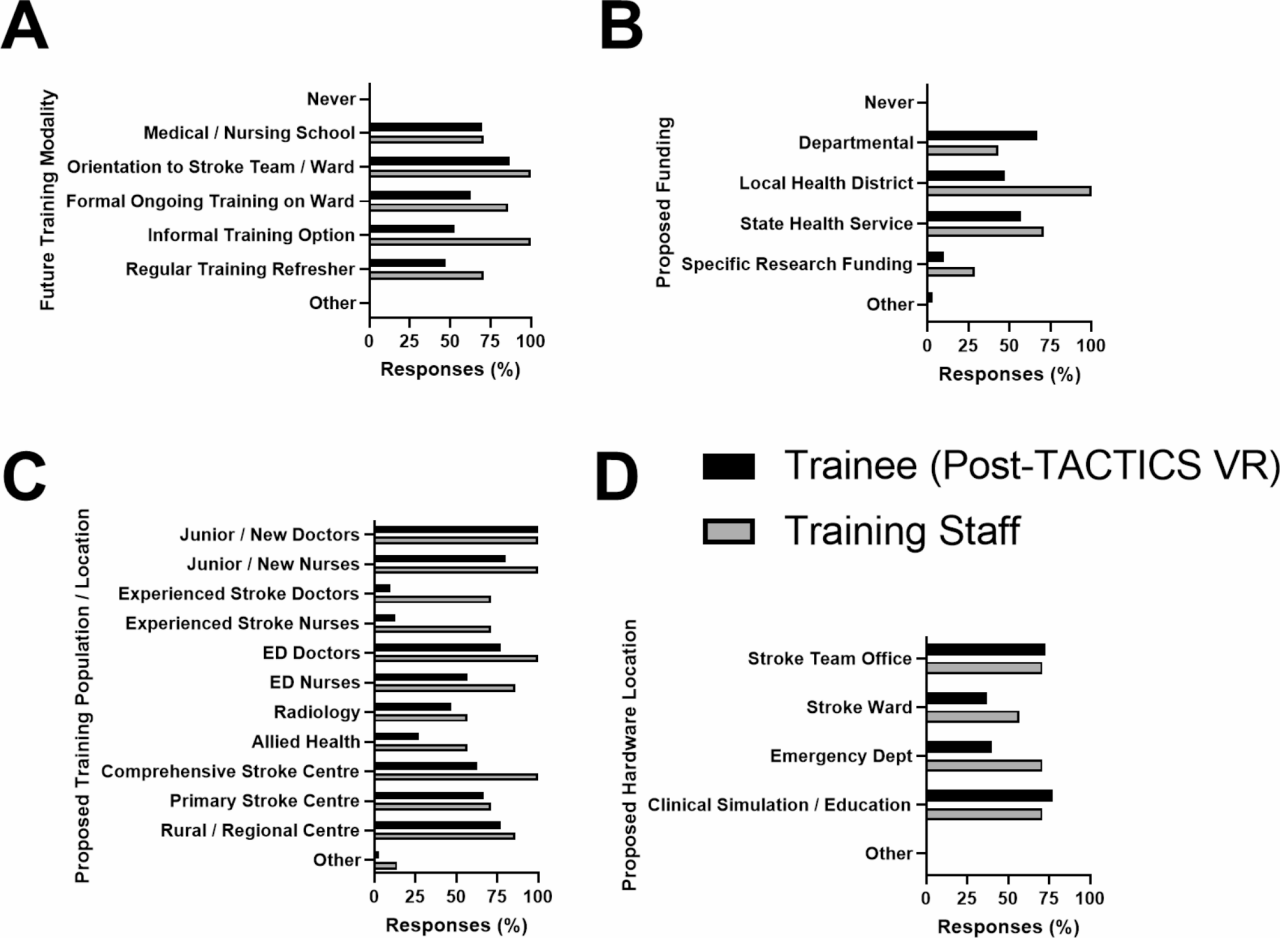
Responses relating to future sustainment approaches for VR training. Potential future (**A**) training modality, (**B**) funding approach, (**C**) target training population and (**D**) VR hardware location. Data presented as proportion of responses noting respondents could select multiple answers. Black bars = trainee post-VR survey; grey bars = training staff survey

Trainees and training staff were also asked how VR training should be delivered in the future. Both populations believed VR training should be used across the range of settings / modalities (Fig. 4A) and supported via a range of funding (Fig. 4B). Training was considered most relevant for junior / new doctors and nurses, followed by ED staff, and less relevant for radiology, allied health or experienced stroke doctors and nurses (Fig. 4C). Further, TACTICS VR training was perceived as being beneficial across a range of clinical settings including in comprehensive stroke centres, primary stroke centres and rural or regional locations (Fig. 4C). Both trainees and training staff suggested VR hardware was best positioned in the stroke team office or at a clinical simulation / education location, and trainees indicated positioning in the stroke ward or ED as less useful (Fig. 4D).

In open-text responses, training staff suggested aspects of the overall VR training implementation approach that were most beneficial: processes and approach (*n* = 6; e.g. “simulation of acute case and feedback on ways to improve”, “group sessions”) and awareness of training content (*n* = 2; e.g. “access to a device”). Training staff also provided areas where the approach could be improved: additional modules / content (*n* = 3; e.g. “multiple scenarios including acute ICH management”), availability (*n* = 2; e.g. “regular scheduling”), practical logistics of multi-team engagement (*n* = 1) or nothing could be improved (*n* = 1) or they were not sure (*n* = 1).

When queried what would assist future sustainment of VR training *at their hospital*, trainees indicated: funding / access (*n* = 9, e.g. “more headsets”), training time allocation (*n* = 8; “specific time allocated”), publicity / awareness of training (*n* = 5) and more modules (*n* = 1). Training staff highlighted: delivery model and integration with existing education (*n* = 5; e.g. “embedding in education curriculum”), training staff buy-in (*n* = 1) or were unsure (*n* = 1). When asked what would assist in sustaining VR training *more broadly* trainees highlighted: funding / access (*n* = 7; e.g. “more availability”), training time (*n* = 4), publicity / awareness (*n* = 3; e.g. “get more people to do and talk about it”) and more modules (*n* = 1). Training staff suggested: making training mandatory (*n* = 3; e.g. “compulsory training for stroke-specific wards”), availability (*n* = 2; e.g. “more available VR units”), more scenarios / developed content (*n* = 2; e.g. “multiple scenarios”) and funding (*n* = 1).

## Discussion

The current study documented perceptions of existing stroke training and integration of VR workflow training, within basic physician training at 2 Australian teaching hospitals from both trainee and training staff perspectives. Perceptions of existing stroke training highlighted areas of unmet need: relatively little reported formal stroke training (median = 10 h over career) and varying views on the suitability of existing approaches. Both respondent populations noted existing barriers to formal stroke training, including factors related to staffing, time and funding. Trainees and training staff both provided positive feedback on VR training in terms of acceptability, usability, perceived training impact and feasibility, with limited issues identified. VR was perceived as an effective training modality and respondents endorsed the future use of VR training and additional module development. The training delivery approach was intentionally flexible and adapted to local constraints over the study period. Areas for improvement to improve uptake and sustainment included factors relating to funding, staffing, awareness and additional content development.

Australian medical trainees receive formal stroke training in medical school and during basic training rotations. The primary focus is on foundational knowledge (e.g. neuroanatomy, pathophysiology, rehabilitation / referral pathways) [5]. However, total hours of formal training are minimal and exposure to acute patient assessment and treatment pathways is variable. Even during advanced neurology training, stroke cases are a subset of the overall portfolio and exposure to hyper-acute management may be limited depending on staffing and processes. Continued education is typically ad hoc and dependent on individual clinician interest in stroke management (e.g. seminar attendance). Of note, trainees reported relatively low levels of formal stroke training time (median = 10-hours total career; 4-hours previous year). This aligns with literature indicating Australian healthcare staff receive limited practical and case-based procedural training in stroke [8]. While the national Australian Stroke Audit highlights the importance of continuing clinical education, it only captures high-level data with no information on the type, extent or suitability of existing training (e.g. 92% of services have a program of continuing education) [4]. Both trainees and training staff highlighted existing barriers to delivery and uptake of formal training, including staffing, time and funding. As such, there is currently an unmet need for effective training to inform best-practice stroke management.

Trainees and training staff provided positive feedback on acceptability, feasibility, usability and perceived training impact of TACTICS VR training specifically, consistent with our previous findings [28, 29, 31]. Further, respondents endorsed the future use of VR in general and TACTICS VR specifically for training and supported additional module development. Viewed through the Technology Acceptance Model [33–35], positive trainee perspectives on both perceived usefulness and ease of use are particularly relevant, as these factors are associated with future adoption of technology. Our findings are consistent with multiple recent reviews assessing VR training in multiple medical professional populations and disease contexts [18–24], which have identified benefits in knowledge development, skills acquisition, confidence, engagement and enjoyment. Importantly, our study addresses several limitations in the existing VR training literature. While most studies only report small pilot studies in controlled settings (e.g. laboratory-based), our study assessed training in a real-world clinical context integrated with existing training with limited additional resources. Further, our study captured the training staff perspective to assess implementation approach and inform future optimisation.

VR training delivery was flexible and feasible with comparable trainee and training staff feedback across both sites, regardless of the specific strategy applied. The SA site deployed a single headset for individual training, which provided flexibility but did require more regular follow-up and communications. In contrast, NSW training occurred in a facilitated group context, which could be specifically scheduled early in rotation and provided an opportunity for discussion and additional training. However, the NSW approach required additional logistics / scheduling, access to multiple VR headsets and coordinating time availability across the trainee cohort. We propose that differing delivery approaches could be tailored to specific contexts and applied in concert to suit local requirements. Both trainees and training staff provided feedback on enablers and barriers to the delivery approach for stroke training in general and specific to the VR modality. Mapping the reported factors onto the Theoretical Domains Framework [38], aspects related to knowledge, skills, beliefs about capabilities, environmental context & resources and social influences. Applying perspectives from the Behaviour Change Wheel [39] we adapted the approach across the study period. For example, we integrated persuasion via mandated training, environmental restructuring to integrate training during orientation, enablement by providing dedicated training time and communication to promote uptake. In future activities, it will be important to include aspects of education, resources / funding, enablement, marketing, service provision, guidelines and clinical pathways to optimise the approach and tailor it to local contexts.

The overall costs were relatively minimal, as the modules were previously developed [28, 29]. For the SA site, minor software modifications were required (<$500 AUD) and a single enterprise-enabled Oculus Quest 2 VR headset was deployed ($1,500 AUD). Implementation occurred within existing quality-improvement activities and did not require specific salary funding. At the NSW site, no software modifications were required and training used an existing fleet of VR headsets owned by the research team. NSW training was delivered by experienced stroke team members, with VR delivery facilitated by a research team member.

We highlight several key strengths of the current study including: deployment outside of a clinical trial; integration within a real-world basic training context with limited additional resources; collection of feedback from both trainee and training staff perspectives at two Australian teaching hospitals; feedback relating to perceptions of existing stroke training approaches and areas of unmet need in general and relating to VR training specifically.

There are several limitations that should be considered when interpreting the study findings. Firstly, the study was limited to two comprehensive stroke training hospitals which may limit the broader validity. Specific local contexts and factors should be considered when applying the learnings within other contexts. Secondly, a pragmatic approach was applied with adaptation of the strategy in response to observations and feedback across the study period. As such, it is not practical to objectively compare the delivery models applied. Thirdly, training effects were only assessed by self-report and no objective assessment of knowledge, skills development and clinical practice outcomes were made. We are currently assessing the effect of TACTICS VR training on clinical practice and treatment metrics in other study contexts. Further, while all trainees completing VR training were encouraged to complete the post-training survey, response rates were not 100% (30 post-training survey responses; 42 total VR sessions; 71% response rate). This introduces potential for selection bias, which should be considered when interpreting results. Finally, we note that training penetration was limited in the flexible SA approach (approximately 60% of the target audience) and it is unclear whether there may be a self-selection bias for participant inclusion. However, 100% uptake occurred with the training cohorts at the NSW site.

## Conclusions

In conclusion, trainees and training staff reported unmet training needs and barriers to existing stroke training at two Australian comprehensive stroke hospitals outside of a formal trial context. Routine VR stroke workflow training was considered acceptable, usable, useful, had perceived training benefits and deployment was feasible. Respondents expressed an interest in receiving VR training in the future, including development of additional cases, modules and extension beyond the stroke field. In line with this feedback, we are currently assessing stroke workflow training modules for nurses and paramedics and expanding training to support cardiac and trauma management. The current study also identified areas for improvement to optimise training uptake, improve integration with existing training and support ongoing sustainment, including funding, awareness and infrastructure.

## Electronic supplementary material

Below is the link to the electronic supplementary material.

Supplementary Material 1: Supplementary table 1. Pre-training trainee survey questions and responses. Supplementary table 2. Post-training trainee survey questions and responses. Supplementary table 3. Training staff survey questions and responses.

## Data Availability

The datasets supporting the conclusions of this article are included within the article and its additional files. Intellectual property for TACTICS VR is owned by The University of Newcastle (Australia). If you are interested in accessing any TACTICS VR application for research purposes, please contact the corresponding author or ATS@newcastle.edu.au.

## References

[1] Saver JL. Time is brain–quantified. Stroke. 2006;37(1):263–6. 16339467 10.1161/01.STR.0000196957.55928.ab

[2] Kunz WG, et al. Public Health and cost benefits of successful reperfusion after Thrombectomy for Stroke. Stroke. 2020;51(3):899–907. 31964289 10.1161/STROKEAHA.119.027874

[3] Kunz W, et al. O-001 lifetime quality of life and cost consequences of treatment delays in endovascular thrombectomy for stroke based on hermes data. J NeuroInterventional Surg. 2018;10(Suppl 2):A1–2.

[4] *National Stroke Audit - Acute Service Report 2023*. 2023, Stroke Foundation: Melbourne Australia.

[5] *Basic Training Curriculum - Adult Internal Medicine*. 2013, The Royal Austalasian College of Physicians.

[6] *Fellowship of the Australasian College for Emergency Medicine Curriculum*. 2024, Australasian College for Emergency Medicine.

[7] *Neurology Advanced Training Curriculum (Adult Medicine Division)*. 2013, The Royal Australiasian College of Physicians.

[8] Paul C, et al. Staff perspectives from Australian hospitals seeking to improve implementation of thrombolysis care for acute stroke. SAGE Open Med. 2019;7:2050312119865656. 31384464 10.1177/2050312119865656 PMC6647204

[9] Meretoja A, et al. Helsinki model cut stroke thrombolysis delays to 25 minutes in Melbourne in only 4 months. Neurology. 2013;81(12):1071. 23946303 10.1212/WNL.0b013e3182a4a4d2

[10] Silsby M, et al. Time to acute stroke treatment in-hours was more than halved after the introduction of the Helsinki Model at Westmead Hospital. Intern Med J. 2019;49(11):1386–92. 30887620 10.1111/imj.14290

[11] Meretoja A, et al. Reducing in-hospital delay to 20 minutes in stroke thrombolysis. Neurology. 2012;79(4):306–13. 22622858 10.1212/WNL.0b013e31825d6011

[12] Forsetlund L et al. Continuing education meetings and workshops: effects on professional practice and health care outcomes. Cochrane Database Syst Rev, 2009(2): p. CD003030. 10.1002/14651858.CD003030.pub2 PMC7138253 19370580

[13] Tahtali D, et al. Implementation of stroke teams and simulation training shortened process times in a regional stroke network-A network-wide prospective trial. PLoS ONE. 2017;12(12):e0188231. 29206838 10.1371/journal.pone.0188231 PMC5716597

[14] Kluge MG, et al. Comparing approaches for selection, development, and deployment of extended reality (XR) teaching applications: a case study at The University of Newcastle Australia. Educ Inf Technol (Dordr). 2023;28(4):4531–62. 36284824 10.1007/s10639-022-11364-2 PMC9584278

[15] Kluge MG, et al. Current state and general perceptions of the Use of Extended reality (XR) technology at the University of Newcastle: interviews and surveys from staff and students. Volume 12. SAGE Open; 2022. p. 21582440221093348. 2.

[16] Kluge MG, et al. Development of a modular stress management platform (performance edge VR) and a pilot efficacy trial of a bio-feedback enhanced training module for controlled breathing. PLoS ONE. 2021;16(2):e0245068. 33529187 10.1371/journal.pone.0245068 PMC7853514

[17] Kluge MG, et al. Evaluation of a virtual reality platform to train stress management skills for a defense workforce: Multisite, mixed methods feasibility study. J Med Internet Res. 2023;25:e46368. 37930751 10.2196/46368 PMC10659241

[18] Dhar E, et al. A scoping review to assess the effects of virtual reality in medical education and clinical care. Digit Health. 2023;9:20552076231158022. 36865772 10.1177/20552076231158022 PMC9972057

[19] Barteit S, et al. Augmented, mixed, and virtual reality-based head-mounted devices for Medical Education: systematic review. Volume 9. JMIR Serious Games; 2021. p. e29080. 3. 10.2196/29080 PMC8299342 34255668

[20] Lie SS, et al. Implementation of virtual reality in Health professions Education: scoping review. JMIR Med Educ. 2023;9:e41589. 36692934 10.2196/41589 PMC9906320

[21] Chen FQ, et al. Effectiveness of virtual reality in nursing education: Meta-Analysis. J Med Internet Res. 2020;22(9):e18290. 32930664 10.2196/18290 PMC7525398

[22] Woon APN, et al. Effectiveness of virtual reality training in improving knowledge among nursing students: a systematic review, meta-analysis and meta-regression. Nurse Educ Today. 2021;98:104655. 33303246 10.1016/j.nedt.2020.104655

[23] Baashar Y, et al. Effectiveness of using augmented reality for training in the Medical professions: Meta-analysis. JMIR Serious Games. 2022;10(3):e32715. 35787488 10.2196/32715 PMC9297143

[24] Bracq MS, Michinov E, Jannin P. Virtual reality Simulation in Nontechnical Skills Training for Healthcare professionals: a systematic review. Simul Healthc. 2019;14(3):188–94. 30601464 10.1097/SIH.0000000000000347

[25] Kuhne C et al. Direct comparison of virtual reality and 2D delivery on sense of presence, emotional and physiological outcome measures. Front Virtual Real. 2023;4.

[26] Wiley E, Khattab S, Tang A. Examining the effect of virtual reality therapy on cognition post-stroke: a systematic review and meta-analysis. Disabil Rehabil Assist Technol. 2020;17(1):50–60. 10.1080/17483107.2020.1755376 32363955

[27] Charles D, et al. Virtual reality design for Stroke Rehabilitation. Adv Exp Med Biol. 2020;1235:53–87. 32488636 10.1007/978-3-030-37639-0_4

[28] Hood RJ et al. Development and pilot implementation of TACTICS VR: a virtual reality-based Stroke Management Workflow Training Application and Training Framework. Front Neurol. 2021;12. 10.3389/fneur.2021.665808 PMC8631764 34858305

[29] Maltby S, et al. TACTICS VR Stroke Telehealth virtual reality training for Healthcare professionals involved in Stroke Management at Telestroke Spoke hospitals: Module design and implementation study. JMIR Serious Games; 2023. 10.2196/43416 PMC10739245 38060297

[30] Ryan A, et al. TACTICS - trial of Advanced CT Imaging and Combined Education support for drip and ship: evaluating the effectiveness of an ‘implementation intervention’ in providing better patient access to reperfusion therapies: protocol for a non-randomised controlled stepped wedge cluster trial in acute stroke. BMJ Open. 2022;12(2):e055461. 10.1136/bmjopen-2021-055461 PMC8845197 35149571

[31] Garcia-Esperon C et al. Rollout of a statewide Australian telestroke network including virtual reality training is associated with improved hyperacute stroke workflow metrics and thrombolysis rate. Front Stroke. 2024;3. 10.3389/fstro.2024.1382608 PMC12802772 41542239

[32] Hall A et al. A bibliographic review of sustainability research output and investment in 10 leading public health journals across three time periods. Public Health Pract. 2023;6:100444. 10.1016/j.puhip.2023.100444 PMC10654021 38028253

[33] Granić A, Marangunić N. Technology acceptance model in educational context: a systematic literature review. Br J Edu Technol. 2019;50(5):2572–93.

[34] Marangunić N, Granić A. Technology acceptance model: a literature review from 1986 to 2013. Univ Access Inf Soc. 2015;14(1):81–95.

[35] Venkatesh V. Determinants of Perceived Ease of Use: integrating control, intrinsic motivation, and emotion into the Technology Acceptance Model. Inform Syst Res. 2000;11(4):342–65.

[36] Brooke J. SUS: a quick and dirty usability scale. Usability Eval Ind. 1995:6.

[37] Witmer BG, Singer MJ. Measuring presence in virtual environments: a presence questionnaire. Presence: Teleoperators Virtual Environ. 1998;7(3):225–40.

[38] Atkins L, et al. A guide to using the theoretical domains Framework of behaviour change to investigate implementation problems. Implement Sci. 2017;12(1):77. 28637486 10.1186/s13012-017-0605-9 PMC5480145

[39] Michie S, van Stralen MM, West R. The behaviour change wheel: a new method for characterising and designing behaviour change interventions. Implement Sci. 2011;6:42. 21513547 10.1186/1748-5908-6-42 PMC3096582

