# Supplementary material for: Implementation and sustainment of virtual reality stroke workflow training for physician trainees at comprehensive stroke centres: a quantitative and qualitative study
Source: BMC Med Educ. 2024 Dec 19;24:1494. doi: 10.1186/s12909-024-06438-3 (PMC11658365; doi:10.1186/s12909-024-06438-3)
Supplement: Supplementary file 1 — Supplementary Material 1: Supplementary table 1. Pre-training trainee survey questions and responses. Supplementary table 2. Post-training trainee survey questions and responses. Supplementary table 3. Training staff survey questions and responses. [file 12909_2024_6438_MOESM1_ESM.docx]

**Implementation and Sustainment of Virtual Reality Stroke Workflow Training for Physician Trainees at Comprehensive Stroke Centres: A Quantitative and Qualitative Study**

Steven Maltby^1,2,3+^, Joshua J. Mahadevan^4,5+^, Neil J. Spratt^2,3,6^, Carlos Garcia-Esperon^3,6^, Murielle G. Kluge^1,2^, Christine L. Paul^3,7^*,* Timothy J. Kleinig^4,5^, Christopher R. Levi^7,8^ & Frederick R. Walker^1,2,3*^

## Supplementary Materials

# Contents

**Supplementary Table 1:** Pre-training trainee survey questions and responses

**Supplementary Table 2:** Post-training trainee survey questions and responses

**Supplementary Table 3:** Training staff survey questions and responses

# Supplementary Tables

**Supplementary Table 1:** Pre-training trainee survey questions and responses

| **Question** | **Response options (n)** |
| --- | --- |
| Q1. What is your specialty area of work? | Emergency Care  Intensive Care  Radiology  Acute Stroke / Neurology (36)  Stroke Rehabilitation  Other (7) - please specify:   - Intern (x3) - Student (x2) - BPT Trainee - General Trainee |
| Q2. What category best describes your employment? | Doctor (e.g. intern / resident medical officer…; 38)  Nurse (e.g. AIN, enrolled nurse…; 1)  Nurse Practitioner  Radiographer  Other (4) - please specify:   - Medical student (x3) - Student |
| Q3. Are you currently involved in a training program (e.g. residency)? | Yes (20)  No (23) |
| Q4. How have you previously received training in stroke management? (check all that apply) | Formal training in medical / nursing school (25)  Formal clinical simulations (6)  Formal training on ward (9)  Via online or face-to-face meetings / seminars (11)  Via printed or online materials (13)  Informal training on the job (27)  Other (2) |
| Q5. How much time have you spent in formal training for stroke management (total career; hours)? | Mean±SD = 122±361  Minimum = 0  25% Percentile = 2  Median = 10  75% Percentile = 50  Maximum = 2000 |
| Q6. How much time have you spent in formal training for stroke management (over the last year; hours)? | Mean±SD = 59±200  Minimum = 0  25% Percentile = 0  Median = 4  75% Percentile = 12  Maximum = 1200 |
| Q7. How frequently do you engage in formal training for stroke management? | One-off training (1)  Weekly (7)  Monthly (3)  Quarterly (2)  Annually (8)  Less than annually (12)  Never (10) |
| Q8. How would you rate the level of current training provision in stroke management at your hospital? | No training available (2)  Insufficient (6)  Adequate (29)  Excellent (6) |
| Q9. What are key enablers to stroke management training at your hospital? | Repeated response category counts:   - Staff / on-site training = 18 - Formal training = 10 - Resources = 5 - Funding = 1 - None = 3   Open-text responses:  South Australia site:   - Consultant-led teaching - Stroke unit-specific informal training (consultants, registrars, stroke nurses) - KPI, daily practice, trials meeting, nurse education sessions - Staffing – Nurse Consultants available to explain code and assist with RMO transition to leading codes independently - Consultant led - Informal teaching from stroke unit whilst employed and working with stroke unit - Great training on the job so far - Doing a stroke rotation - Daily stroke management - Availability coinciding with opportunities - Online resources, individual stroke consultants and registrars - Formalised training and feedback, radiology meetings - Formal training (x2 responses) - Formal teaching - Formalised training processes (x2) & college training - Formalised training and resources to enable - Formal orientation and training - Resources including guidelines, stroke nurses - Resources - Online resources & protocols - Nil - N/A (x2)   New South Wales site:   - Informal teaching on the ward or stroke call from motivated specialists - Informal on the job teaching watching consultant / fellow run stroke calls - Consultants on the job - Senior staff knowledge; resources; processes - Good supportive bosses; good sharing; funding - Formal session arranged for us |
| Q10. What are existing barriers or areas of unmet need for stroke management training at your hospital? | Repeated response category counts:   - Time = 9 - Staffing = 6 - Stroke process complexity / practicality = 5 - Resources = 3 - Funding = 2 - None (6) or unsure (4) = 10   Open-text responses:  South Australia site:   - Time (x3) - Specific time allocation for training - Time available in working hours - Time resource - Too busy to engage - Introduction to code stroke prior to commencement of rotation - Training of stroke patient flow at a systems level - If you don’t do a stroke rotation there is limited exposure to formal teaching - Processes, staffing - Difficult with starting on nights to get formal orientation for running codes without nearby senior support (other than via phone) - Resources - Resources – no formal training for juniors - Staffing - Funding for formal sessions - N/A (x3) - No barriers - Not identified - Nil - Unsure (x2) - Not sure   New South Wales site:   - Staffing on the wards - Staffing - Chronically understaffed - Time; staffing - Resources, funding, time, workload - No formal training for SRMO - Unsure |
| Q11. Approximately how many acute stroke patients have you cared for or treated over the course of your career? (Please estimate) | Less than 10 patients (10)  11-20 patients (5)  21-30 patients (3)  31-40 patients (3)  41 patients or more (17)  Not applicable to my practice (5) |
| Q12. I am confident in my knowledge around accessing telehealth for acute stroke assessment or treatment. | Strongly agree  Agree  Undecided  Disagree  Strongly disagree  Not applicable  (Mean = 2.9 ± 1.0, n=43; Strongly agree = 5) |
| Q13. I am aware of local stroke management criteria and processes. | Strongly agree  Agree  Undecided  Disagree  Strongly disagree  Not applicable  (Mean = 3.6 ± 1.0, n=43; Strongly agree = 5) |
| Q14. I am confident in my ability to effectively assess or treat acute stroke patients. | Strongly agree  Agree  Undecided  Disagree  Strongly disagree  Not applicable  (Mean = 3.1 ± 1.0, n=43; Strongly agree = 5) |
| Q15. I am confident in my ability to optimally communicate with my colleagues to enable effective treatment of acute stroke patients. | Strongly agree  Agree  Undecided  Disagree  Strongly disagree  Not applicable  (Mean = 3.3 ± 1.0, n=43; Strongly agree = 5) |
| Q16. I am confident in my understanding of workflow practices to effectively manage acute stroke patients. | Strongly agree  Agree  Undecided  Disagree  Strongly disagree  Not applicable  (Mean = 3.2 ± 1.2, n=43; Strongly agree = 5) |
| Q17. I am confident in my ability to make improvements to how acute stroke care is provided to patients presenting to this hospital. | Strongly agree  Agree  Undecided  Disagree  Strongly disagree  Not applicable  (Mean = 3.0 ± 1.2, n=X; Strongly agree = 5) |
| Q18. Have you previously used any of the TACTICS VR training modules (check all that apply)? | Yes – TACTICS VR Hyperacute Stroke Treatment (1)  Yes – TACTICS VR Telestroke  Yes- TACTICS VR Stroke Nursing  Yes – Not certain which module (2)  No (40) |
| Q19. What is your experience with virtual reality technology? (This may include gaming and home use as well as other virtual simulation experiences – please provide as much detail as possible) | Over 100 hours of usage (1)  50-99 hours of usage  10-49 hours of usage (4)  Less than 10 hours of usage (15)  No experience – I have never used VR or similar technology (23) |
| Q20. I believe that VR can be an effective method to teach or transfer knowledge about stroke management. | Strongly agree  Agree  Undecided  Disagree  Strongly disagree  Not applicable  (Mean = 3.9 ± 0.8, n=43; Strongly agree = 5) |
| Q21. I would use VR-based training if it were available at my hospital. | Yes – only if mandated / required (11)  Yes – voluntarily (32)  No |
| Q22. If VR-based training (e.g. 20-minute training module in clinical offices) were available at my hospital I would be. | More likely to engage in training (22)  Equally likely to engage in training (20)  Less likely to engage in training (1) |
| Q23. If VR training were directly linked with continuing professional development (e.g. CPD credits) I would be more likely to complete training. | Strongly agree  Agree  Undecided  Disagree  Strongly disagree  Not applicable  (Mean = 3.9 ± 1.0, n=43; Strongly agree = 5) |
| Q24. I feel confident in regards to managing the technical aspect of the VR training tool. | Strongly agree  Agree  Undecided  Disagree  Strongly disagree  Not applicable  (Mean = 3.1 ± 1.2, n=43; Strongly agree = 5) |
| Q25. Are you prone to motion sickness? | Yes (12)  No (31) |
| Q26. What do you hope to learn from the TACTICS VR stroke training? | Repeated response category counts:   - Stroke management aspects = 22 - VR technology use = 7 - Fun / anything = 2 - Unsure = 1   Free-text responses:  South Australia site:   - Acute stroke management (x3) - How to manage code stroke (x2) - How to approach stroke - Code stroke hyper-acute decision making - Stroke management - More competence in acute stroke presentations - Increased proficiency and efficiency in assessing acute stroke - A better system for swift code running and obtaining pertinent information - Protocol & process - How to approach an active stroke patient with a clear plan - Improve knowledge of acute stroke management - More confident in running code stroke - Learning to use VR and acute stroke management - Familiarity with the Code Stroke process and environment - Dealing with the technology - How to use this resource - Simulated learning experience - Practical experience in safe environment - Fun experience - Anything - Competency in stroke identification and management   New South Wales site:   - Increased confidence and efficiency - Become more confident running a stroke call as the AT / in charge i.e. leadership under stress - Acute stroke assessment - Improvement in skills - Practicing simulated environments of clinical cases - Unsure |

VR = virtual reality; Data presented as mean +/- SD.

**Supplementary Table 2:** Post-training trainee survey questions and responses

| **Question** | **Response options (n)** |
| --- | --- |
| Q1. The VR hardware (e.g. headset and controller) were comfortable and easy to use. | Strongly agree  Agree  Undecided  Disagree  Strongly disagree  Not applicable  (Mean = 3.9 ± 1.1, n=30; Strongly agree = 5) |
| Q2. The TACTICS VR user interface was straight-forward and easy to use (e.g. menu system, buttons, etc). | Strongly agree  Agree  Undecided  Disagree  Strongly disagree  Not applicable  (Mean = 4.3 ± 0.9, n=30; Strongly agree = 5) |
| Q3. I enjoyed TACTICS VR training. | Strongly agree  Agree  Undecided  Disagree  Strongly disagree  Not applicable  (Mean = 4.6 ± 0.6, n=30; Strongly agree = 5) |
| Q4. TACTICS VR – provided useful information. | Strongly agree  Agree  Undecided  Disagree  Strongly disagree  Not applicable  (Mean = 4.7 ± 0.5, n=30; Strongly agree = 5) |
| Q5. TACTICS VR provided accurate information. | Strongly agree  Agree  Undecided  Disagree  Strongly disagree  Not applicable  (Mean = 4.6 ± 0.5, n=30; Strongly agree = 5) |
| Q6. TACTICS VR was an effective tool for transferring knowledge about stroke management practices. | Strongly agree  Agree  Undecided  Disagree  Strongly disagree  Not applicable  (Mean = 4.5 ± 0.7, n=30; Strongly agree = 5) |
| Q7. TACTICS VR improved my awareness of stroke management approaches | Strongly agree  Agree  Undecided  Disagree  Strongly disagree  Not applicable  (Mean = 4.5 ± 0.6, n=30; Strongly agree = 5) |
| Q8. TACTICS VR improved my understanding of stroke management approaches. | Strongly agree  Agree  Undecided  Disagree  Strongly disagree  Not applicable  (Mean = 4.5 ± 0.6, n=30; Strongly agree = 5) |
| Q9. TACTICS VR increased my confidence in stroke management workflow. | Strongly agree  Agree  Undecided  Disagree  Strongly disagree  Not applicable  (Mean = 4.5 ± 0.7, n=30; Strongly agree = 5) |
| Q10. TACTICS VR was of sufficient realism to communicate the critical aspects of workflow. | Strongly agree  Agree  Undecided  Disagree  Strongly disagree  Not applicable  (Mean = 4.4 ± 0.7, n=30; Strongly agree = 5) |
| Q11. The feedback provided at the end of the VR training module was constructive and useful. | Strongly agree  Agree  Undecided  Disagree  Strongly disagree  Not applicable  (Mean = 4.3 ± 0.7, n=30; Strongly agree = 5) |
| Q12. Did you feel motion sick or nauseous while participating using TACTICS VR? | Yes – I could not compete the training due to motion sickness  Yes – But I was able to complete training (6)  No (24) |
| Q13. Should TACTICS VR training be used for stroke management training? | Yes (26)  No (1)  Unsure (3) |
| Q14. In what context should TACTICS VR training be provided? (select all that apply) | Never  Medical / Nursing School (21)  At Orientation to Stroke Team / Ward / ED (26)  Formal Ongoing Training on Ward / ED (19)  Informal Training Option (e.g. in-service training) (16)  Regular Refresher of Training (14)  Other – please specify: |
| Q15. How should TACTICS VR training be funded? (select all that apply) | Never  Departmental Funding (20)  Local Health District (14)  State Health Service (17)  Specific Research Funding (3)  Other (1) – please specify: |
| Q16. Who would gain benefit from participating in TACTICS VR training? (select all that apply) | Junior / New Doctors (30)  Junior / New Nurses (24)  Experienced Stroke Doctors (3)  Experienced Stroke Nurses (4)  Emergency Department Doctors (23)  Emergency Department Nurses (17)  Radiology Staff (14)  Allied Health Staff (8)  Staff at Comprehensive Stroke Centres (19)  Staff at Primary Stroke Centres (20)  Staff at Rural / Regional Centres (23)  Other (1) – please specify:   - Medical Students |
| Q17. Where should TACTICS VR training hardware be kept to promote uptake? (select all that apply) | Stroke Team Office (22)  Stroke Ward (11)  Emergency Department (12)  Clinical Simulation / Education Facility (23)  Other – please specify: |
| Q18. What elements of the TACTICS VR training were most beneficial? | Repeated response category counts:   - Training content = 18 - VR modality / technology = 8   Free-text responses:  South Australia site:   - Feedback about choices made and pressure with clocks at the top of the screen - Carrying out the process in a sequential order - Up to date decision making pathways - [Expert’s] teachings (talking head videos) - Familiarity with potential situation - Information provided - Realistic examples and process training - Videos explaining as you go what to focus on and how to structure running your code - Low pressure helpful questions - Interactive choices for e.g. assessment and radiology - Being able to have freedom in choosing what to do allowing you to make realistic mistakes - The ongoing feedback - Explanation of imaging - Explanations and walkthroughs - Realism - No performance anxiety - Simulated scenarios - Realistic. Engaging   New South Wales site:   - Running through a realistic sim with NOK [next of kin] questions - Decision-making - Thinking about how to improve efficiency - Practicing in safe setting; immediate feedback - Active learning vs. passive; gamification to help provide incentive + time pressure / urgency - Safe space to run through a stroke call - Realistic; choice of options; ongoing feedback |
| Q19. What elements of TACTICS VR training could be improved? | Repeated response category counts:   - Specific content = 10 - User experience = 8 - Hardware = 2 - More scenarios = 1 - Not applicable = 1   Free-text responses:  South Australia site:   - More education about treatment decision process - Realistic timeflow, making penalties accrue at the end of the module rather than the start - I would request early alteplase based on the plain CTB appearance - Site specific information - Could do more complex clinical cases - Be good to include going through a NIHSS - When penalties are highlighted at the end to show a breakdown of missed sections - Length of explanations; post acute stroke management; could be training centre-specific - Was not clear that could skip sections that weren’t relevant; controls not explained very well in the game - Realism - Intuitiveness of acquiring information from surrounding - Less commentary or teaching throughout – useful but distracting and gives you less chance to try it all in one go - More comfortable headset - VR headset – motion sickness - More scenarios - Honestly can’t think of much, it was an excellent scenario   New South Wales site:   - Check for specific contraindications (e.g. uncontrolled hypertension); check for stroke mimics (e.g. BGL) - The running timer is distracting - Perhaps less interruptions by speakers + more active decision-making - Setup - More complicated for senior clinicians |
| Q20. Should additional modules for TACTICS VR stroke training be developed?  Q21. If yes, what modules would be most useful? | Yes (27)  No (3)  Repeated response category counts:   - Stroke (additional cases; other stroke types; presentations) = 12 - Stroke mimics =2 - Stroke assessment = 2 - Other clinical needs =3   Free-text responses:  South Australia site:   - Varying encounters or negative code strokes - Various stroke syndromes and management of ICH - Difference scenarios; Haemorrhage, CT negative, lacunar - Vague symptoms that may or may not need full stroke series (i.e. how to deal with non-clear-cut codes) - Reperfusion therapy post-stroke care - Treatment decision and stroke assessment details - Stroke management but with added twists or complications e.g. haemorrhage identified on CT or strokes not suitable for thrombolysis or clot retrieval etc. - Other strokes / mimics - Varied presentations of stroke and stroke mimics - Completing a NIHSS - MR interpretation; Rehab decision making - Sepsis, STEMI - No it would be too long - As above.   New South Wales site:   - Haemorrhagic stroke treatment - Other stroke scenarios - Remote stroke call - STEMI calls |
| Q22. Did you experience any issues that prevented you from completing TACTICS VR training?  Q23. If yes, please describe: | Yes (4)  No (26)  Repeated response category counts:   - Time = 3 - Hardware = 2 - User error = 1 - Funding = 1   Free-text responses:  South Australia site:   - Pressures with current patient load and very little time during work day set aside for training - Difficult to secure time while managing ward duties - Doesn’t work well with glasses and I couldn’t find the extension tube for glasses, nor the settings to change the screen for use without glasses - No issues but difficult to adjust headset suitably for clear vision   New South Wales site:   - Funding + allocated training time - Accidentally went back to start |
| Q24. What would assist with sustaining TACTICS VR training at your hospital? | Repeated response category counts:   - Funding / Access = 9 - Time = 8 - Publicity / Awareness = 5 - More modules = 1   Free-text responses:  South Australia site:   - Protected time for training - Dedicated protected time - Funding, rostering to complete the training - Funding I suppose! Access to headsets and time in which to do this training - Reminders; specific time allocated - More headsets - Definitely funding. And an accepted standard of training modules that can easily be shared between state, etc. - N/A - maybe affordability - Greater publicity - Awareness that it exists - Engagement with medical students - As above – more modules   New South Wales site:   - Rostered, protected, mandatory sessions - More time allocated during day to complete it - Funding; dedicated mandatory training time - Funding (x3) - Awareness that it exists |
| Q25. What would enable TACTICS VR training to be sustained overall more broadly? | Repeated response category counts:   - Funding / Access = 7 - Time = 4 - Publicity / Awareness = 3 - More modules = 1   Free-text responses:  South Australia site:   - More medical school training - Freedom for implementation as per each individual unit - Definitely funding. And an accepted standard of training modules that can easily be shared between states, etc. - More availability - Specific time allocated - Protected time for training and more modules - Greater awareness that it is readily available already - incorrect perception that it is beyond scope of current practice - Get more people to do and talk about it - Awareness and availability to access   New South Wales site:   - Funding (x2) - Dedicated staffing - Protected sessions - More time allocated during day to complete it - Dedicated mandatory training |
| Q26. I am confident in my knowledge around accessing telehealth for acute stroke assessment or treatment. | Strongly agree  Agree  Undecided  Disagree  Strongly disagree  Not applicable  (Mean = 4.0 ± 0.7, n=30; Strongly agree = 5) |
| Q27. I am aware of local stroke management criteria and processes. | Strongly agree  Agree  Undecided  Disagree  Strongly disagree  Not applicable  (Mean = 4.1 ± 0.8, n=30; Strongly agree = 5) |
| Q28. I am confident in my ability to effectively assess or treat acute stroke patients. | Strongly agree  Agree  Undecided  Disagree  Strongly disagree  Not applicable  (Mean = 4.0 ± 0.6, n=30; Strongly agree = 5) |
| Q29. I am confident in my ability to optimally communicate with my colleagues to enable effective treatment of acute stroke patients. | Strongly agree  Agree  Undecided  Disagree  Strongly disagree  Not applicable  (Mean = 4.1 ± 0.6, n=30; Strongly agree = 5) |
| Q30. I am confident in my understanding of workflow practices to effectively manage acute stroke patients. | Strongly agree  Agree  Undecided  Disagree  Strongly disagree  Not applicable  (Mean = 4.3 ± 0.6, n=30; Strongly agree = 5) |
| Q31. I am confident in my ability to make improvements to how acute stroke care is provided to patients presenting to this hospital. | Strongly agree  Agree  Undecided  Disagree  Strongly disagree  Not applicable  (Mean = 4.1 ± 0.8, n=30; Strongly agree = 5) |

VR = virtual reality; Data presented as mean +/- SD.

**Supplementary Table 3:** Training staff survey questions and responses

| **Question** | **Response options (n)** |
| --- | --- |
| Q1. What is your specialty area of work? | Emergency Care  Intensive Care  Radiology  Acute Stroke / Neurology (7)  Stroke Rehabilitation  Other - please specify: |
| Q2. What category best describes your employment? | Doctor (e.g. intern / resident medical officer…; 4)  Nurse (e.g. AIN, enrolled nurse…; 3)  Nurse Practitioner  Radiographer  Other - please specify: |
| Q3. How long have you been in this role? | < 1 year  1-5 years (2)  6-10 years  > 10-years (5) |
| Q4: How long have you worked with assessment or treatment of acute stroke patients? | < 1 year  1-5 years  6-10 years (1)  > 10-years (6) |
| Q5: Who do you usually train in stroke management? | Doctor Trainee (4)  Doctor (e.g. resident medical officer, staff specialist…) (4)  Nurse Trainee  Nurse (e.g. AIN, enrolled nurse…) (4)  Nurse Practitioner (2)  Radiographer (1)  Management (e.g. heads of department, clinical leaders…)  Other – please specify: |
| Q6: How many people have you trained in stroke management (estimate number annually)? | Mean±SD = 55±53  Minimum = 4  25% Percentile = 5  Median = 50  75% Percentile = 100  Maximum = 150 |
| Q7: How many people have you trained in stroke management (estimate number total career)? | Mean±SD = 541±679  Minimum = 40  25% Percentile = 50  Median = 400  75% Percentile = 600  Maximum = 2000 |
| Q8: How much time do you spend delivering formal training for stroke management annually (hours)? | Mean±SD = 82±119  Minimum = 0  25% Percentile = 10  Median = 25  75% Percentile = 200  Maximum = 300 |
| Q9. How frequently do you engage in formal training for stroke management? | One-off training  Weekly (2)  Monthly (1)  Quarterly (2)  Annually (1)  Less than annually (1) |
| Q10: How have you provided training in stroke management? | Formal training in medical / nursing school (3)  Formal clinical simulations (3)  Formal training on ward (6)  Via online or face-to-face meetings / seminars (5)  Via printed or online materials (2)  Informal training on the job (6)  Other – please specify: |
| Q11: What are key enablers to stroke management training at your hospital? | Repeated response category counts:   - Formal training = 3 - Staff / on-site training = 3 - Resources = 2   Open text responses:  South Australia site:   - Formal training at start of junior staff rotations, mortality / morbidity meetings, separation summary and neuro-radiology meetings - Formal time for training (scheduled); multiple training devices; positive feedback and review of junior doctors who have enjoyed the training - Hyperacute stroke management with KPIs in our organisation-wide instruction manual; We use a novice-to-expert framework which incorporates outside agency resources (e.g. ASNEN, Stroke Foundation, Angels Initiative)   New South Wales site:   - Good relations with ED and other teams to be trained - Stroke training that is included in already running program, good working relationship with emergency department - Enthusiasm of trainers and potential trainees. Fear of getting it wrong and looking a fool. - Formalised training resources |
| Q12: What are existing barriers or areas of unmet need for stroke management training at your hospital? | Repeated response category counts:   - Time = 4 - Staffing = 4 - Resources = 2   Open text responses:  South Australia site:   - Time constraints with clinical workload - Relieving junior staff starting ad hoc during term, new consultant staff - Resources and staffing; issues with the ability to get more than 3 nurses on the stroke unit to attend a face-to-face workshop on a given day; it relies on piece-meal education   New South Wales site:   - Time, time and time! Difficult to capture all the teams / relevant teams. - Time for staff to attend education – resources - Finding time for trainers, and to get people together. Lack of dedicated staff to help organize. Training is always less urgent than clinical need. - Staffing & processes |
| Q13: New staff (e.g. doctors, nurses) typically have sufficient clinical knowledge in stroke management upon their arrival at our hospital. | Strongly agree  Agree  Undecided  Disagree  Strongly disagree  (Mean = 2.4 ± 1.1, n=7; Strongly agree = 5) |
| Q14: New staff typically have a good understanding of stroke workflow upon their arrival at our hospital. | Strongly agree  Agree  Undecided  Disagree  Strongly disagree  (Mean = 2.1 ± 1.1, n=7; Strongly agree = 5) |
| Q15: New staff typically are aware of local stroke management criteria and processes. | Strongly agree  Agree  Undecided  Disagree  Strongly disagree  (Mean = 2.4 ± 1.0, n=7; Strongly agree = 5) |
| Q16: New staff typically can effectively assess or treat acute stroke patients. | Strongly agree  Agree  Undecided  Disagree  Strongly disagree  (Mean = 2.3 ± 1.1, n=7; Strongly agree = 5) |
| Q17: New staff typically delay stroke management decisions unnecessarily, due to lack of familiarity with processes. | Strongly agree  Agree  Undecided  Disagree  Strongly disagree  (Mean = 3.4 ± 1.0, n=7; Strongly agree = 5) |
| Q18: New staff typically can optimally communicate with their colleagues to enable effective treatment of acute stroke patients. | Strongly agree  Agree  Undecided  Disagree  Strongly disagree  (Mean = 3.1 ± 0.9, n=7; Strongly agree = 5) |
| Q19: New staff typically have a good understanding of effective workflow practices to manage acute stroke patients. | Strongly agree  Agree  Undecided  Disagree  Strongly disagree  (Mean = 2.3 ± 0.8, n=7; Strongly agree = 5) |
| Q20: New staff typically have the ability to make improvements to how acute stroke care is provided to patients presenting to this hospital. | Strongly agree  Agree  Undecided  Disagree  Strongly disagree  (Mean = 3.1 ± 1.2, n=7; Strongly agree = 5) |
| Q21: What are the biggest areas of concern in stroke management training / skills at your hospital? | Repeated response category counts:   - Staffing = 6 - Processes = 2   Open text responses:  South Australia site:   - When new staff begin without prior introduction to local hospital stroke workflow - Junior doctor fear, anxiety and apprehension at attending CODE strokes - Capture the new RMOs before they start their rotation, then we give them huge amounts of information and off they go   New South Wales site:   - Turnover - Sustainability, given that training in the hyperacute phase is delivered by our team. Hopefully in the future, the training will be undertaken by supporting department staff - Very small pool of potential trainers, who are all overcommitted. Very ad hoc at present. - Process |
| Q22: I believe that VR can be an effective method to teach or transfer knowledge about stroke management. | Strongly agree  Agree  Undecided  Disagree  Strongly disagree  (Mean = 4.1 ± 0.7, n=7; Strongly agree = 5) |
| Q23: I feel confident in regards to managing the technical aspects of the VR training tool. | Strongly agree  Agree  Undecided  Disagree  Strongly disagree  (Mean = 4.1 ± 0.7, n=7; Strongly agree = 5) |
| Q24: Should TACTICS VR training be used for stroke management training? | Yes (7)  No  Unsure |
| Q25: In what context should TACTICS VR training be used? | Never  Medical / Nursing School (5)  At Orientation to Stroke Team / Ward / ED (7)  Formal Ongoing Training on Ward / ED (6)  Informal Training Option (e.g. in-service training) (7)  Regular Refresher of Training (5)  Other – please specify: |
| Q26: How should TACTICS VR training be funded? | Never  Departmental Funding (3)  Local Health District (7)  State Health Service (5)  Specific Research Funding (2)  Other – please specify: |
| Q27: Who would benefit from participating in TACTICS VR training? | Junior / New Doctors (7)  Junior / New Nurses (7)  Experienced Stroke Doctors (5)  Experienced Stroke Nurses (5)  Emergency Department Doctors (7)  Emergency Department Nurses (6)  Radiology Staff (4)  Allied Health Staff (4)  Staff at Comprehensive Stroke Centres (7)  Staff at Primary Stroke Centres (5)  Staff at Rural / Regional Centres (6)  Other (1) – please specify:   - More targeted training would be good for others too |
| Q28: Where should TACTICS VR training hardware be kept to promote uptake? | Stroke Team Office (5)  Stroke Ward (4)  Emergency Department (5)  Clinical Simulation / Education Facility (5)  Other – please specify: |
| Q29: What elements of the TACTICS VR implementation approach were most beneficial? | Repeated response category counts:   - Processes & approach = 6 - Awareness / access = 2   Open text responses:  South Australia site:   - Simulation of acute case and feedback on ways to improve - Mandating TACTICS VR training - Knowing what it involves   New South Wales site:   - It was good for us to think on the material to include and having a think about what we wanted to teach to the teams - Availability and approach - Group sessions - Access to a device & training |
| Q30: What elements of the TACTICS VR implementation approach could be improved? | Repeated response category counts:   - Additional modules / content = 3 - Availability = 2 - Logistical challenges = 1 - Nothing / Not sure = 2   Open text responses:  South Australia site:   - Multiple scenarios including acute ICH management. Sub-acute stroke management scenario where lysis is contraindicated - I didn’t do well with it as it didn’t use the same decision-making process as me in a code stroke process - Wider accessibility   New South Wales site:   - Regular scheduling. Dedicated packages for different craft groups (e.g. radiographers / allied health play quite different roles) - I feel that is challenging to keep the local teams to be using in ED. ED might be too busy for VR and VR might work better in more protocol-driven scenarios (e.g. pre-hospital or even medical school) - Nothing - Not sure |
| Q31: Should additional modules for TACTICS VR stroke training be developed?  Q32: If yes, what modules would be most useful? | Yes (5)  No (2)  Repeated response category counts:   - Stroke (additional cases; other stroke types; presentations; audiences) = 4 - Other clinical needs = 1   Free-text responses:  South Australia site:   - As above - Post-care for EVT patients, blood pressure management, managing patient deterioration geared at nurses   New South Wales site:   - General stroke care – ASSIST, positioning, communication - Radiographers / allied health - Pre-hospital; Safe discharge of stroke patient |
| Q33: Did you experience any issues that prevented trainees from completing TACTICS VR training?  Q34: If yes, please describe: | Yes (3)  No (4)  Repeated response category counts:   - Time = 2 - Engagement = 2   Free-text responses:  South Australia site:   - Trainee time constraints, balancing clinical responsibilities. Difficult to motivate them to participate initially. - Engaging them to undertake it. ED staff not likely to engage as we have 24-hour stroke team   New South Wales site:   - Too busy with their clinical roles |
| Q35: What would assist with sustaining TACTICS VR training at your hospital? | Repeated response category counts:   - Model / integration = 5 - Buy-in = 1 - Not sure = 1   Free-text responses:  South Australia site:   - Mandatory training sessions - Formal scheduled VR training time - If I like it more, all assessors of medical staff expect it as part of their orientation   New South Wales site:   - Embedding in education curriculum. - Implementing the VR training into Education Days - Regular scheduled sessions each term - That’s a challenge. Not sure. |
| Q36: What would enable TACTICS VR training to be sustained overall more broadly (e.g. at the health district / state / national level)? | Repeated response category counts:   - Requirement = 3 - Availability = 2 - More scenarios / content = 2 - Funding = 1   Free-text responses:  South Australia site:   - Requirement training much like hand hygiene to help provide safe optimal care - More available VR units, multiple scenarios - We only had one, so to use it in a workshop environment was not practical with 60 participants   New South Wales site:   - Compulsory training for stroke-specific wards - VR being included as part of mandatory Education Days or sessions - The technology is there, but it would be more used if this is used not just for stroke but also for other conditions (e.g. AMI, trauma…) - Funding for package and for trainers |
